# Transcriptome analysis of *Kluyveromyces marxianus* under succinic acid stress and development of robust strains

**DOI:** 10.1007/s00253-024-13097-3

**Published:** 2024-04-09

**Authors:** Du-Wen Zeng, Yong-Qiang Yang, Qi Wang, Feng-Li Zhang, Mao-Dong Zhang, Sha Liao, Zhi-Qiang Liu, Ya-Chao Fan, Chen-Guang Liu, Lin Zhang, Xin-Qing Zhao

**Affiliations:** 1 Key Laboratory of Microbial Metabolism, Joint International Research Laboratory of Metabolic & Developmental Sciences, School of Life Sciences and Biotechnology, Shanghai Jiao Tong University, Shanghai, 200240 China; 2 SINOPEC Dalian Research Institute of Petroleum and Petrochemicals Co., Ltd, Dalian, 116045 China; 3 School of Life Sciences, Hainan University, Haikou, 570228 China

**Keywords:** *Kluyveromyces marxianus*, Succinic acid (SA), SA tolerance, SA responsive gene, Transcriptome analysis

## Abstract

**Abstract:**

*Kluyveromyces marxianus* has become an attractive non-conventional yeast cell factory due to its advantageous properties such as high thermal tolerance and rapid growth. Succinic acid (SA) is an important platform molecule that has been applied in various industries such as food, material, cosmetics, and pharmaceuticals. SA bioproduction may be compromised by its toxicity. Besides, metabolite-responsive promoters are known to be important for dynamic control of gene transcription. Therefore, studies on global gene transcription under various SA concentrations are of great importance. Here, comparative transcriptome changes of *K. marxianus* exposed to various concentrations of SA were analyzed. Enrichment and analysis of gene clusters revealed repression of the tricarboxylic acid cycle and glyoxylate cycle, also activation of the glycolysis pathway and genes related to ergosterol synthesis. Based on the analyses, potential SA-responsive promoters were investigated, among which the promoter strength of *IMTCP2* and *KLMA_50231* increased 43.4% and 154.7% in response to 15 g/L SA. In addition, overexpression of the transcription factors Gcr1, Upc2, and Ndt80 significantly increased growth under SA stress. Our results benefit understanding SA toxicity mechanisms and the development of robust yeast for organic acid production.

**Key points:**

*• Global gene transcription of *K. marxianus* is changed by succinic acid (SA)*

*• Promoter activities of IMTCP2 and KLMA_50123 are regulated by SA*

*• Overexpression of Gcr1* *,* *Upc2, and Ndt80 enhanced SA tolerance*

**Graphical Abstract:**

**Figure:**
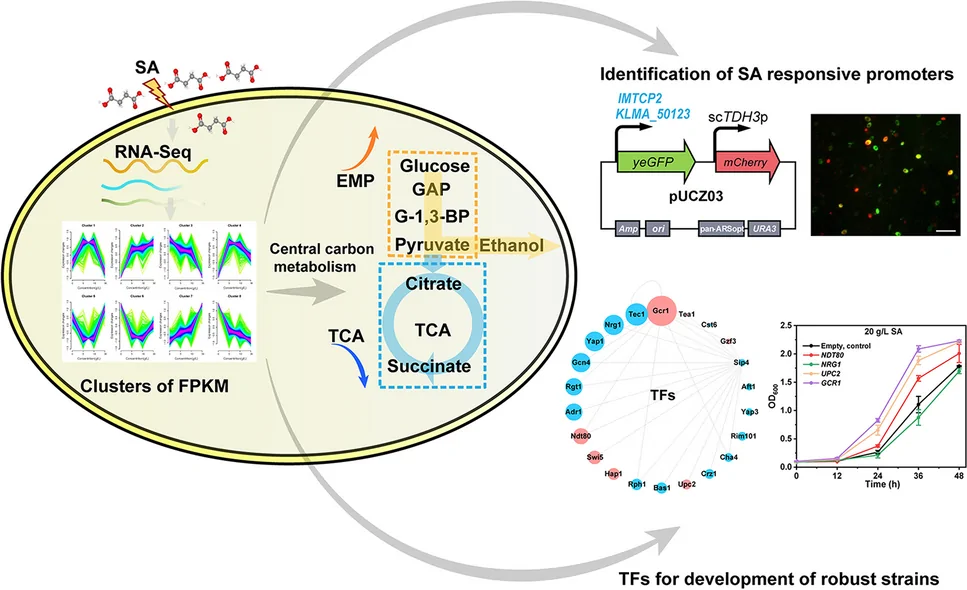

**Supplementary Information:**

The online version contains supplementary material available at 10.1007/s00253-024-13097-3.

## Introduction

*Kluyveromyces marxianus* is a non-conventional yeast which is generally recognized as safe (GRAS). *K. marxianus* has become a valuable cell factory for various biotechnological applications (Karim et al. 2020) due to various advantages: Firstly, *K. marxianus* grows rapidly, and is the fastest growing eukaryote (Groeneveld et al. 2009); secondly, the yeast is also famous for high thermotolerance (Banat et al. 1992). Moreover, *K. marxianus* can utilize various carbon sources, such as glucose, xylose, and inulin (Do et al. 2019; Fonseca et al. 2013). Last but not least, *K. marxianus* is genetically amenable, which makes it an attractive yeast cell factory. *K. marxianus* has been used to produce industrial enzymes, fuel ethanol, single-cell proteins, vaccines, the flavoring compound 2-phenylethanol, and lactic acid (Qiu et al. 2023; Zhou et al. 2018). For efficient production, it is required that the microbial strains are tolerant to various stress conditions that are relevant to industrial applications, which include high product concentration, low pH, or high temperature (Zhao et al. 2016; Zhang et al. 2023). Therefore, it is of great interest to develop stress tolerant *K. marxianus* strains for economic bioproduction.

The depletion of fossil resources and the resulting climate changes have been the driving force behind the rapid development of the bio-based economy. Production of biofuels, platform chemicals, and material precursors from renewable biomass using microbial strains are of great importance for the bioeconomy (Liu et al. 2020). Yeast cell factories have received increasing attention for the production of biodegradable plastics using organic acids as monomers, such as lactic acid and succinic acid (SA) (Zhang et al. 2023). Due to decreased cellular activity during the production of organic acids, a pH neutralizer is generally used (Ullah et al. 2012; Sun et al. 2023). For efficient organic acid production, it is highly desired that the microbial strains are tolerant to low pH (Liu et al. 2023; Sun et al. 2023; Tran et al. 2023).

As a building block chemical and platform chemical, SA is widely used in material production, and also in the production of surfactants, detergents, and pharmaceuticals (Li et al. 2021). To achieve economic SA production, high titer and productivity are desired. However, high concentrations of SA may lead to intracellular acidification and metabolic dysfunction, which may be similar to the effects of other weak organic acids (Mira et al. 2010; Tretter et al. 2016). In addition to low pH stress (Ribeiro et al. 2022), the toxicity of SA molecules may be also an important toxic factor like other weak organic acids. However, to date, there are no reports on SA response or toxicity in yeast.

Dynamic regulation of gene transcription is desired for fine-tuning gene expression levels to achieve delicate control in metabolic engineering (Hartline et al. 2021). For example, the development of an L-malic acid-responsive promoter to control the transcription levels of key transcription factors (TFs) for increasing malic acid tolerance and production (Liang et al. 2021). The production of lactic acid and itaconic acid was increased approximately tenfold and fivefold respectively by using the pH-inducible promoter (Rajkumar et al. 2016; Yin et al. 2017). However, to date, no studies have been reported on SA-responsive promoters in microbial strains.

Although tolerance to several stresses such as ethanol, high temperature, and inhibitors from lignocellulosic hydrolysate have been studied in *K. marxianus* (Diniz et al. 2017; Kosaka et al. 2022; Wang et al. 2018), the response to SA and development of SA tolerant *K. marxianus* have not been reported. In this study, we performed a comparative RNA-seq analysis of *K. marxianus* treated with different concentrations of SA and analyzed the global changes in gene expression to identify the SA-responsive promoters and potential gene targets for SA tolerance. These results provide a strategy for the study of metabolite response mechanisms in yeast and also benefit the development of robust yeast with organic acid resistance.

## Materials and methods

### Strains and medium

The haploid strain *K. marxianus* NBRC1777 (NBRC, Japan) was used as the host. The disruption and overexpression of genes in *K. marxianus* were achieved by CRISPR/Cas9-mediated genome editing based on the previous report (Rajkumar et al. 2019). *Escherichia coli* DH5α (TransGen Biotech Co., Ltd, Beijing, China) was used as the host for plasmids construction. Yeast transformation was performed using the lithium acetate transformation method (Lyu et al. 2021). The strains harbored the candidate promoter evaluation system characterized by the ratio of green and red fluorescence intensity on the centromeric plasmid and contained the TFs overexpression cassette on the episomal plasmid. More details of construction including the plasmids schematic diagram are described in the supplementary information (Fig. S1a, Fig. S2). All strains, and plasmids used in this study are listed in Supplementary Table S1, and the primers are listed in Supplementary Table S2.

Yeast extract peptone dextrose (YPD) (20 g/L glucose, 10 g/L yeast extract, 20 g/L peptone) contained different final concentrations (0, 5, 10, and 30 g/L) of SA to obtain YPDS medium, noting that 60 g/L SA stock solution was first filter sterilized, and then mixed with semi-finished YPD to make the YPDS medium. The preparation of Synthetic complete (SC) medium minus uracil (SC-ura) was performed based on the previous work (Zeng et al. 2020).

### Yeast culture condition

*K. marxianus* NBRC1777 was inoculated (initial OD_600_ approximately 0.1) in a YPDS medium containing different concentrations of SA (0, 5, 10, and 30 g/L respectively) for RNA isolation. The yeast strain was cultured at 37 °C with shaking at 200 rpm for 6 h until the cells were harvested. Then, the culture broth was centrifuged, washed, and centrifuged again at 4 °C to obtain cell bullets.

### RNA sequencing and data processing

The isolated total RNA samples were sent to Genergy Bio-Technology Co., Ltd. (Shanghai, China) for quality and quantity evaluation of cDNA, which was then sequenced using the Illumina HiSeq 4000 instrument (Illumina, San Diego, CA, USA). The *K. marxianus* DMKU3-1042 genome sequence information (NCBI accession number: PRJDA65233) (Lertwattanasakul et al. 2015) was used as the reference genome for the assembly of the clean reads. The transcript abundance was normalized and represented by the FPKM (fragments per kilobase of exon model per million mapped fragments).

### Transcriptome data analysis

The FPKM values of a specific gene transcription level at four different SA concentrations were clustered by using R package “Mfuzz” (version 3.2.3), which is a soft-clustering algorithm based on fuzzy c-means (Kumar and Futschik 2007). According to the trend of FPKM values, the transcript level changes of all genes were classified into different gene clusters, then for the next analysis including gene ontology (GO) and metabolic pathway (KEGG) finally visualized by using the R package “ClusterProfiler” (version 3.8) (Yu et al. 2012) *p* values were adjusted using Benjamini and Hochberg method (Benjamini and Hochberg 1995), and *p* value < 0.05 was set as the threshold.

### Prediction of potential TFs by analysis of promoter sequences

Based on the promoter sequence information of the target gene using the genome sequence of *K. marxianus* DMKU3-1042 as the reference and the knowledge of the conservation between TFs and their DNA binding motifs of *S. cerevisiae* strain S288C (Monteiro et al. 2019), the potential TFs of specific genes in *K. marxianus* strain NBRC1777 were predicted using the YEASTRACT + database (http://yeastract-plus.org/ncyeastract/kmarxianus/).

### Quantitative real-time PCR assay

Briefly, the gene transcription level was analyzed by quantitative PCR (qPCR) on the CFX Connect™ Optics Module (BIO-RAD, Hercules, CA, USA) PCR instrument as described in previous reports (Zhang et al. 2019). The *ACT* gene (*KLMA_70051* coding for actin protein) as the reference and all the qPCR primers used in this study are listed in Supplementary Table S2.

### Cell fluorescence intensity analysis and candidate promoter activity evaluation

Cells were cultured in SC medium minus uracil (SC-ura) (pH 4.67), SC-ura with 15 g/L SA (pH 2.73, 0.13 M), SC-ura without SA (pH 2.73 adjusted by hydrochloric acid), and SC-ura with 0.13 M other organic acid (lactic/citric/malic/fumaric/pyruvic/α-ketoglutaric acid) and harvested at mid-exponential phase, washed twice with ice-cold 10 mmol/L phosphate buffer (PBS, pH 7.0) and resuspended in PBS. Samples of 1 × 10^4^ cells were monitored through the FITC (fluorescein isothiocyanate) and PE (phycoerythrin) channels for fluorescent detection (Gump and Thorburn 2014) via a flow cytometer (Cytoflex S, Beckman Coulter, Brea, CA, USA) and analyzed with the FlowJo™ 10.10.0 software (FlowJo software, Ashland, OR, USA). Fluorescence pictures of cells were captured through a fluorescence microscope (ECHO, RVL-100-G, San Diego, CA, USA).

### Metabolite determination

Extracellular metabolites in the fermentation broth, such as succinic acid, glucose, ethanol, acetic acid, and glycerol were measured by high-performance liquid chromatography (HPLC) as described in our previous work (Ye et al. 2022).

## Results

### SA inhibits the growth of *K. marxianus* with the increasing concentration

**Fig. 1:**
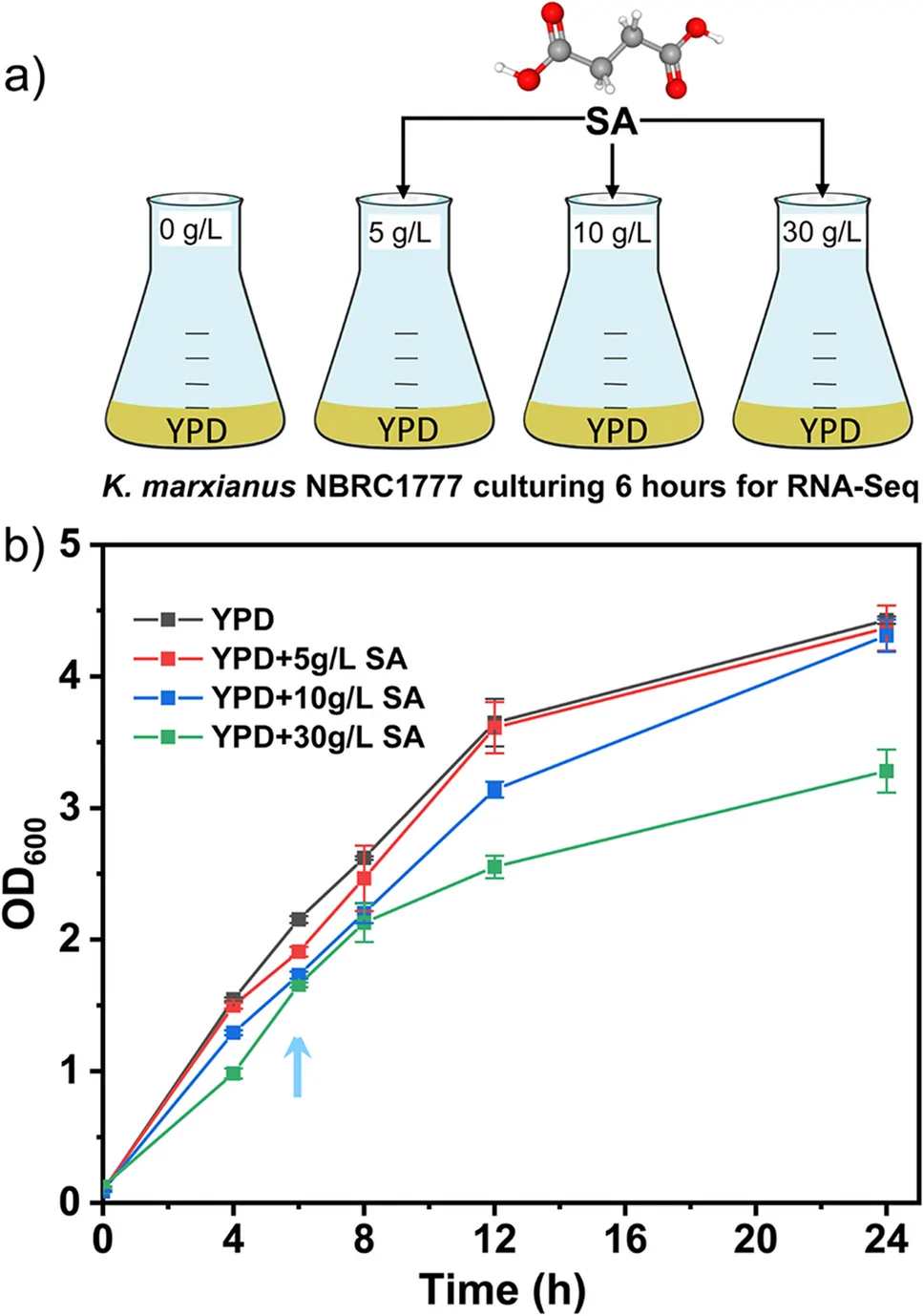
Growth of *K. marxianus* strain NBRC1777 under different SA concentrations. **a** The strains were cultured in YPD medium with different concentrations (0, 5, 10, and 30 g/L) of SA in flasks covered by oxygen permeable membrane at 37 °C and 200 rpm with triple independent parallel experiments performed. **b** The black, red, blue, and green squares and lines represent YPD medium containing 0, 5, 10, and 30 g/L SA, respectively. The horizontal axis represents the culture time (in hours) and the vertical axis represents the optical absorption density OD_600_. Strains were harvested at the 6-h time point (blue arrow) for transcriptome analysis

To find out the dynamic SA responsive genes, we cultured the strain NBRC1777 in YPD medium with 0, 5, 10, and 30 g/L SA respectively for 6 h. It can be seen that the growth was more inhibited at higher SA concentrations. The biomass of strain NBRC1777 was reduced by 3.3%, 7.8%, and 22.1%, respectively (Fig. 1). The samples were harvested at mid-log phase for RNA-seq analysis at 6 h (Fig. 1, blue arrow).

### Different gene clusters responding to different concentrations of SA

**Fig. 2:**
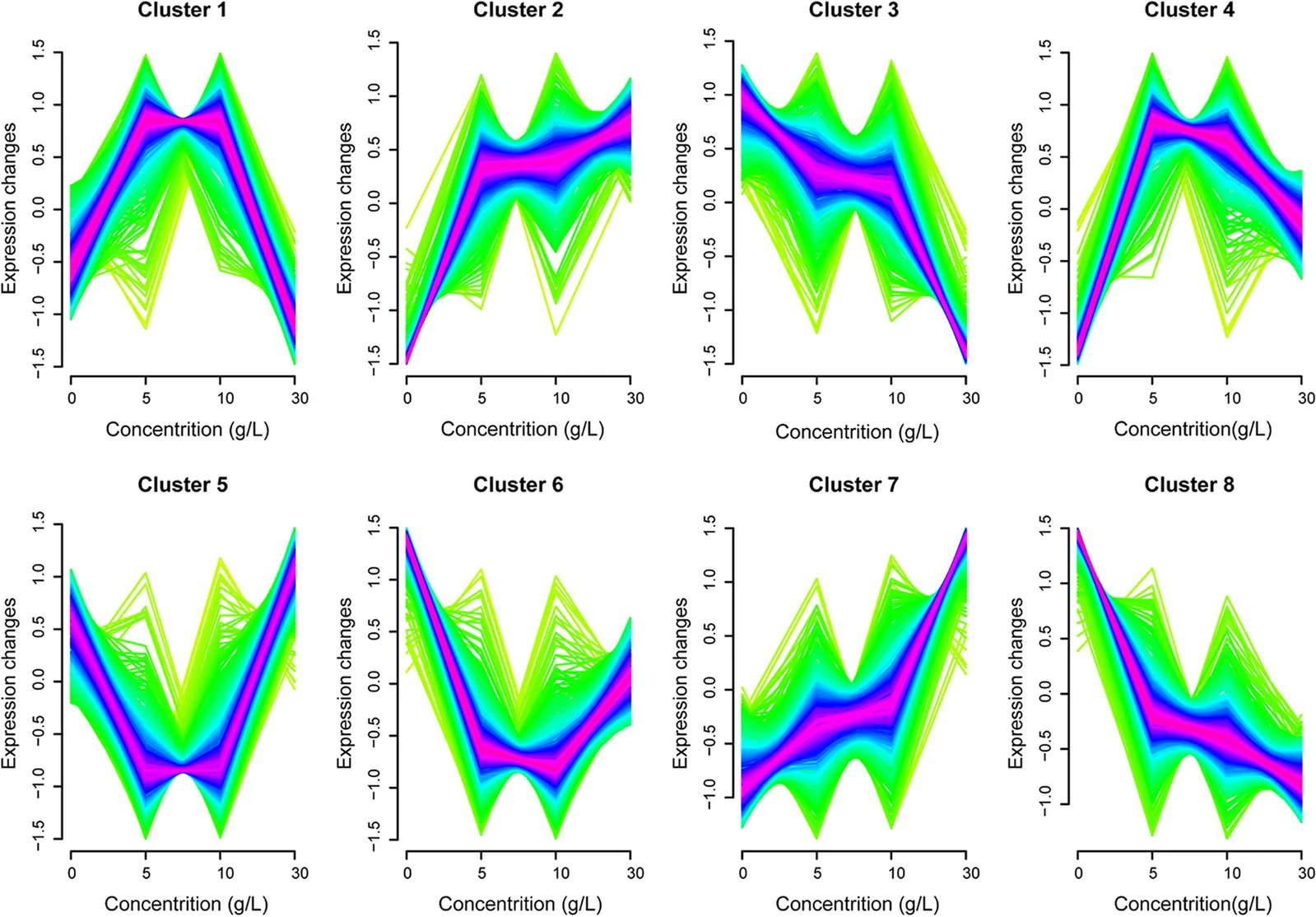
Different patterns of dynamic gene transcription profiles when *K. marxianus* NBRC1777 was exposed to different concentrations of SA. Expression profiles of eight clusters as determined by corresponding FPKMs (fragments per kilobase of exon model per million mapped fragments) of each gene at four different SA concentrations, were clustered by using the Mfuzz R package. Genes with high support (indicated by a high fuzziness score) are displayed in purple, while those with weak support are in green within the cluster. The histogram displays the SA concentration on the horizontal axis and gene expression change on the vertical axis. Genes in clusters 2 and 7 exhibit increased transcription levels with higher SA concentrations, whereas genes in clusters 3 and 8 display decreased transcription levels

To analyze the effect of transcription level from strains NBRC1777 on the increasing concentrations of SA, the gene expression level that was characterized by FPKM value was estimated. We manually considered all the detected genes, regardless of whether they showed significant changes or not. The FPKM values were integrated into gene clusters classification according to the principle of fuzzy mean using R package “Mfuzz” (version 3.2.3) (Futschik and Carlisle 2005) (Fig. 2). In different clusters, the gene cluster 2_7 (Fig. 2, cluster 2 and 7) indicated that the transcription level is positively correlated with the increased concentration of SA, and the gene cluster 3_8 (Fig. 2, cluster 3 and 8) that is negatively correlated with the increasing SA concentration. Interestingly, other gene clusters exhibited varying patterns of transcription level changes, and the scientific inquiries underlying these trends merit future exploration.

### Cluster profile analysis of SA-responsive genes in *K. marxianus*

**Fig. 3:**
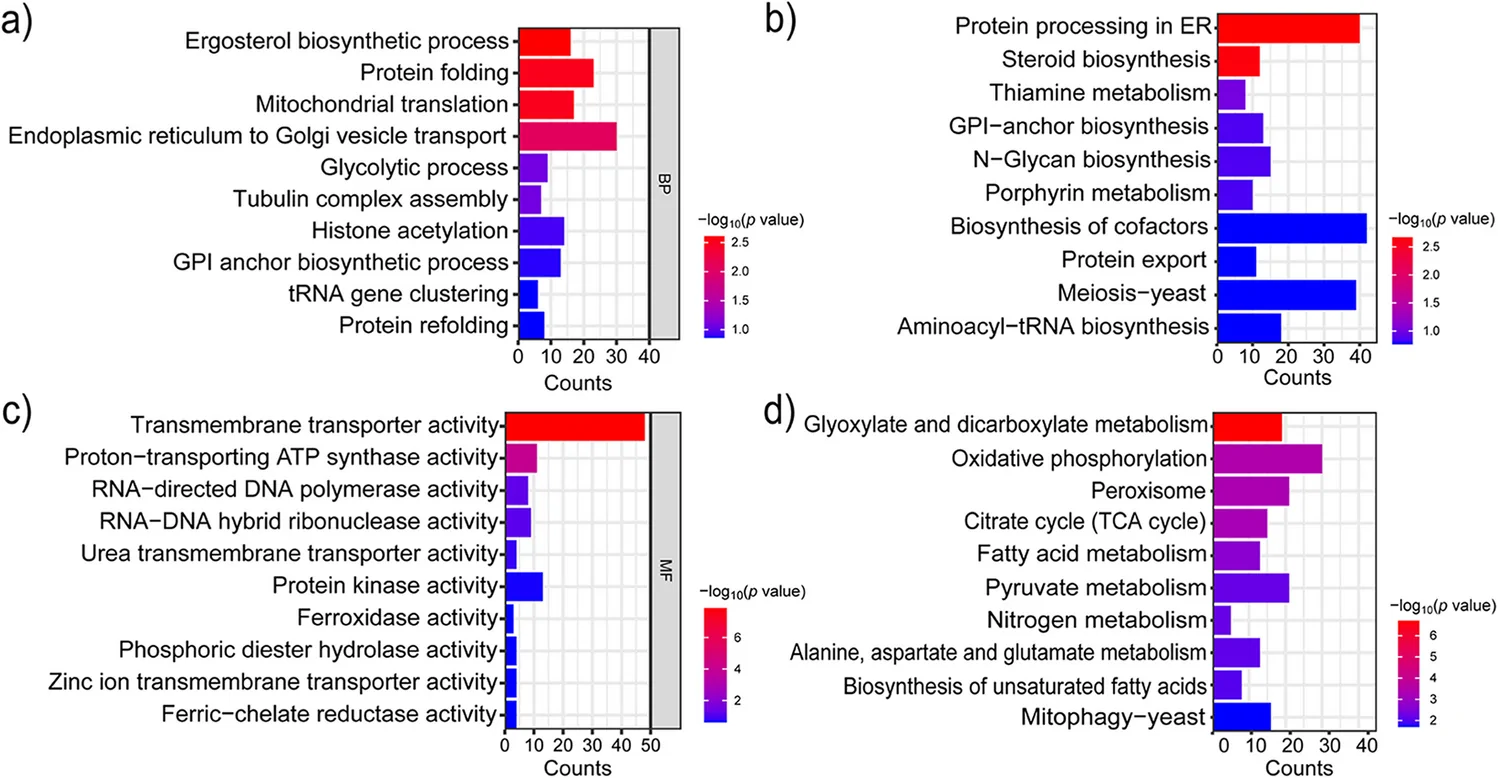
Enrichment analysis for global transcription responses to SA in *K. marxianus*. The analysis was performed on the genes belonging to cluster 2_7 and cluster 3_8. (**a**, **c**) The categorizations of genes based on their biological processes and molecular functions are represented. (**b**, **d**) represent the KEGG enrichment analysis of genes belonging to cluster 2_7 and cluster 3_8. The counts of genes are shown on the x-axis, and the GO and KEGG terms description are shown on the y-axis

To ensure an in-depth analysis of all the transcribed genes in *K. marxianus*, a reliable self-constructed database of global genes involved was built for basic enrichment analysis (Supplementary Table S3). The data of gene names corresponding to terms including KEGG and GO (Supplementary Table S4) were collected from the websites (https://rest.kegg.jp/link/pathway/kmx; https://www.uniprot.org/id-mapping), then analyzed and visualized by using the R package “ClusterProfiler” (version 3.8) (Yu et al. 2012). We also performed GO (BP) enrichment analysis on the gene cluster (cluster 2_7). The value of -log10 (*p*.adjust) ≥ 1.3 was used to represent the significant level of enrichment in this study. The GO enrichment analysis is shown in Fig. 3a, the ergosterol biosynthesis (GO:0006696), protein folding (GO:0006457), and protein translation in mitochondria (GO:0032543) were found to be changed significantly. The KEGG enrichment analysis shown in Fig. 3b revealed that the pathway of protein processing in the endoplasmic reticulum (path: kmx04141), steroid biosynthesis (path: kmx00100), and thiamine metabolism (path: kmx00730) pathways were significantly enriched (*p*.adjust < 0.05).

The GO enrichment analysis of 1267 genes on the gene clusters (cluster 3_8) was performed, and the results are shown in Fig. 3c; transcriptionally downregulated genes were significantly enriched in transporter activity (GO:0022857). After KEGG enrichment, analysis of cluster 3_8 is shown in Fig. 3d, the downregulated genes were significantly enriched in glyoxylate and dicarboxylic acid metabolism (kmx00630), as well as in oxidative phosphorylation (kmx00190), which are closely related to SA metabolism.

### Effects of different concentrations of SA on transcription of central carbon metabolism genes

**Fig. 4:**
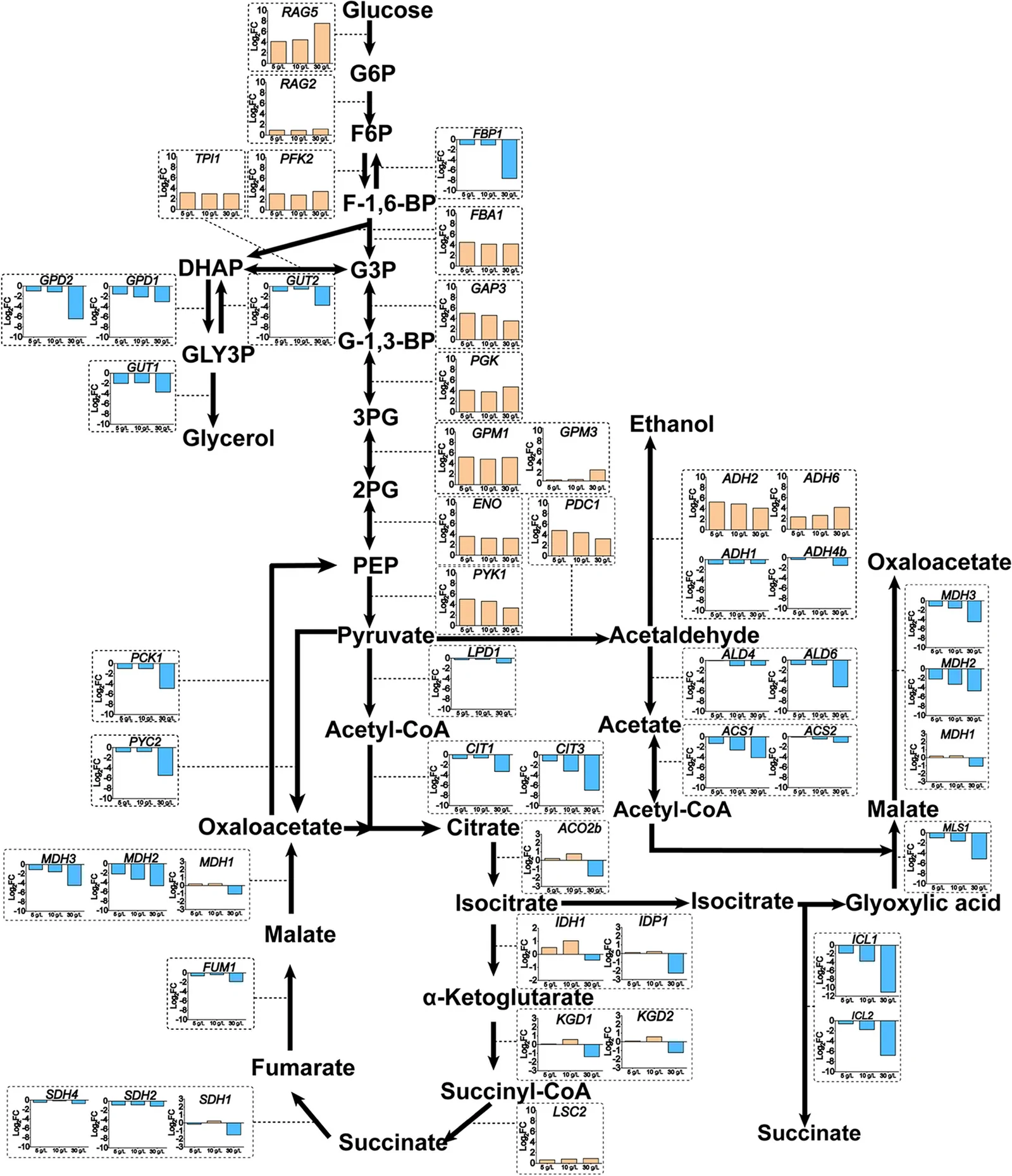
Effects of SA addition on transcription changes of genes involved in central carbon metabolism in *K. marxianus*. The histograms illustrate the relative transcription level fold change (log_2_FC values, y-axis) of genes when strains were exposed to different concentrations (x-axis) of SA. The blue bars indicate downregulation of gene transcription, and the orange bars indicate upregulation compared to the SA-free condition (YPD medium only). *RAG5*, hexokinase; *RAG2*, glucose-6-phosphate isomerase; *PFK2*, 6-phosphofructokinase 1; *FBP1*, fructose-1,6-bisphosphatase I; *FBA1*, fructose-bisphosphate aldolase; *TPI1*, triosephosphate isomerase; *GPD1/2*, glycerol-3-phosphate dehydrogenase; *GUT1/2*, glycerol kinase; *GAP3*, glyceraldehyde-3-phosphate dehydrogenase; *PGK*, phosphoglycerate kinase; *GPM1*/3, phosphoglycerate mutase; *ENO*, enolase; *PYK1*, pyruvate kinase; *LPD1*, dihydrolipoyl dehydrogenase; *CIT1*/*3*, citrate synthase; *ACO2b*, aconitate hydratase; *IDH1/IDP1*, isocitrate dehydrogenase; *KGD1/2*, 2-oxoglutarate dehydrogenase E1 component; *LSC2*, succinyl-CoA ligase subunit β; *SDH1/2/4*, succinate dehydrogenase; *FUM1*, fumarate hydratase; *MDH1/2/3*, malate dehydrogenase; *PYC2*, pyruvate carboxylase; *PCK1*, phosphoenolpyruvate carboxykinase; *ADH1/2/4/6*, alcohol dehydrogenase; *ALD4/6*, aldehyde dehydrogenase; *ACS2*, acetyl-coenzyme A synthetase 2; *MLS1*, malate synthase; *ICL1/2*, isocitrate lyase

Through the overall analysis of the transcriptome data, we found that genes in the major pathways of energy sources, including the glycolytic pathway and the tricarboxylic acid (TCA) cycle, were significantly changed by SA addition. As shown in Fig. 4, many genes of the glycolytic pathway were upregulated, such as the critical genes *RAG5* and *PFK2* (Fig. 4). The expression levels of genes involved in ethanol production, such as *ADH2* and *ADH6*, were also significantly upregulated. Many genes of the TCA pathway were downregulated, such as *CIT1*, and *CIT3* coding mitochondrial citrate synthase, which are rate-limiting enzymes of the TCA cycle. In addition, the key gene isocitrate lyase *ICL1* in the glyoxylate pathway, which is directly involved in SA production was also downregulated. From these results, it is speculated that the SA could inhibit the glyoxylate pathway and the TCA cycle leading to energy deficiency under the SA stress conditions, which pushes the flux increase of the glycolytic pathway and promotes the ability of ethanol production. This deduction was supported by the *K. marxianus* NBRC1777 culture experiment which was repeated under different concentrations of SA. The metabolites were detected by HPLC and analysis found that the ethanol production per unit strain increased 44.6% up to 3.97 g/(L·OD_600_) for 8-h culture (Supplementary Fig. S3a); meanwhile, the revolved important gene transcription levels measured by qPCR (Supplementary Fig. S3b) were consistent with our expectation, which also enhanced the reliability of the speculation.

### Analysis of promoters responding to different concentrations of SA

The promoters are crucial genetic elements that could be used not only for strain construction but also to find revolved TFs to illustrate regulation mechanisms. In order to dig out SA response promoters, we analyzed and selected three types of promoters including (1) transcription level not changed, (2) increased and (3) decreased with SA treatment. A total of 36 promoters were selected from the gene cluster 2_7 and cluster 3_8 according to the standard deviation change range shown in Supplementary Fig. S1b.

Furthermore, 13 promoters were randomly selected from the 36 promoters for SA response verification assay by the green and red dual fluorescent protein expression system. The red fluorescent protein mCherry is expressed under the control of a constitutive promoter *scTDH3* from *Saccharomyces cerevisiae*, while the green fluorescent protein yeGFP is expressed under the control of the promoter of interest (Supplementary Fig. S1a). Thus, the expression level of *mCherry* is relatively constant as the reference to eliminate the interference of the fluorescence background caused by the change of plasmid copy number in *K. marxianus*. The promoter strength of candidate promoters controlling *yeGFP* expression could be characterized by the ratio of the green fluorescence intensity to the red fluorescence intensity in cells. The promoter *IMTCP2* (alias *NC1*, *KLMA_40174*) (Kumar et al. 2021; Ye et al. 2022) in the plasmid marked with the 5-phosphate orotate gene was replaced by several candidate promoters respectively, and the corresponding plasmids were constructed (Supplementary Table S1, line 5 to line 17). Then, all those plasmids harboring new different promoters (1000 bp upstream of the native genes) were transferred into uracil auxotrophic strains of *K. marxianus* ZW01 (NBRC1777, *ura3∆*) to obtain the series of strains listed in Supplementary Table S1 (line 30 to line 42). The positive control utilized the strain ZW01 (NBRC1777, *ura3∆*) carrying the plasmid featuring the *IMTCP2* promoter, while the test group comprised other strains featuring corresponding candidate promoters.

**Fig. 5:**
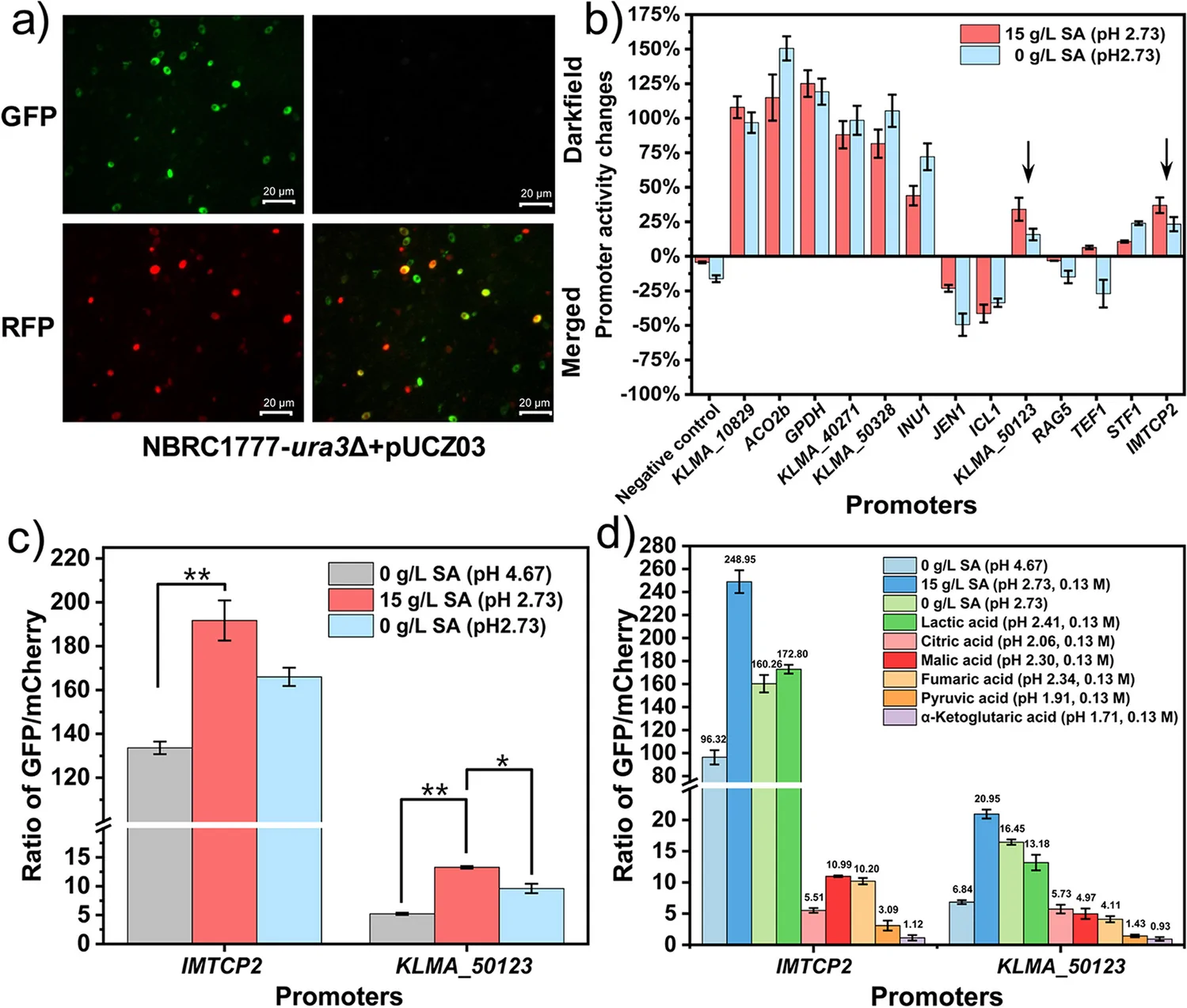
SA-responsive promoter mining and characterization. **a** Candidate and sc*TDH3* promoters control the expression of the fluorescent proteins yeGFP and mCherry, respectively. **b** The promoter strength is represented by the ratio of green and red fluorescence intensity, while the changes in promoter strength are characterized by the parameter of promoter activity change. Activity change of candidate promoters under 15 g/L SA (pH 2.73) or without SA and pH adjusted to 2.73 by hydrochloric acid, the black arrow means stronger response to SA than corresponding pH. **c** The ratio of yeGFP and mCherry fluorescence intensity of the promoters *IMTCP2* and *KLMA_50123* with and without SA. **d** Response of promoters to other organic acids at the same molar concentration as SA when cells were cultured in SC-ura medium for 9 h at 37 °C and 200 rpm. Student *t*-test was used for statistical analyses with significant levels, *: *P* < 0.05, **: *P* < 0.01

All those strains containing green and red fluorescence (Fig. 5a) were cultured in SC-ura medium with 0 g/L SA (pH 4.67), 15 g/L SA (pH 2.73), and 30 g/L SA (pH 2.73 adjusted by 11.9 M of HCl). The promoter strength is represented by the ratio of green and red fluorescence intensity, while the changes in promoter strength are characterized by the parameter of promoter activity change. The fluorescence intensity was tested by a flow cytometer. As shown in Fig. 5b, under the stimulation of 15 g/L SA, the promoter strength of P_IMTCP2_ and P_KLMA_50123_ increased, that of P_ICL1_ decreased, and that of P_TEF1_ was regarded as unchanged (Fig. 5b, Supplementary Fig S1c). The promoter strength of P_IMTCP2_ increased by 43.3% and 24.3%, that of promoter P_KLMA 50123_ by 154.7% and 83.7% respectively, under 15 g/L SA and its pH 2.73 (Fig. 5c), indicating that the two promoters were SA responsive. In addition, we also tested whether the promoters (P_IMTCP2_, P_KLMA 50123_) that respond to other organic acids at the same molar concentration (lactic/citric/malic/fumaric/pyruvic/α-ketoglutaric acid), the strain grew up and had the stronger promoter activity in the 0.13 M lactic acid than that in SC-ura medium without SA (pH 4.67) (Fig. 5d).

### Identification of TFs for enhancing SA tolerance

**Fig. 6:**
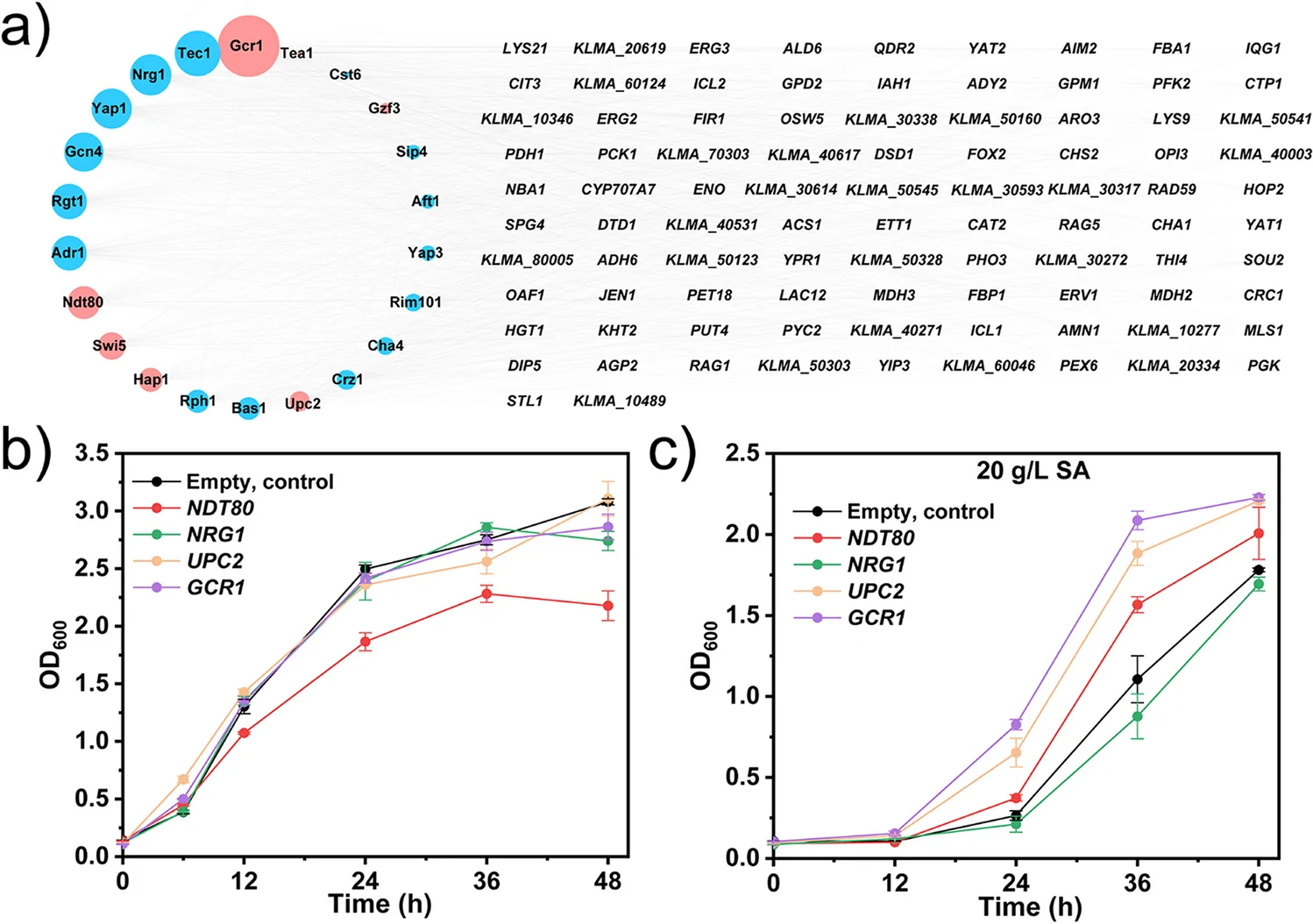
Overexpressing transcription factors (TFs) enhanced SA resistance. (a) The TFs identified from the transcriptome data (left, circles) are compared to those predicted from the promoter sequences of the top 100 genes with the greatest changes in transcription levels (right). The circle area on the left grows larger as the number of genes (on the right) regulated by the TFs (left) increases. Red denotes up-regulation of gene transcription by the corresponding TFs while blue denotes down-regulation. (b, c) Growth assay to evaluate the property of SA resistance of strains overexpressed different TFs on the plasmid. Strains harbored plasmids were cultured in SC-ura medium with or without 20 g/L SA at 37 °C and 200 rpm

In order to find the TFs that regulate SA tolerance, we analyzed the potential TFs of the top 100 genes with the most significant transcription level change. We assume that transcription changes in the TFs would lead to changes of their regulated genes. The involved TFs were predicted according to the promoter sequence information in the website http://yeastract-plus.org, in which 94 of the top 100 genes were successfully identified and 126 TFs were predicted. Concurrently, a total of 90 TFs clustered in cluster 2_7 and cluster 3_8 with transcriptional fold changes Log_2_ FC ≥ 1 was screened out from our transcription omics data (Supplementary Table S3). Subsequently, the 126 predicted TFs were aligned with the 90 screened out from the transcriptomic data. Finally, the 21 TFs were obtained that regulated the expression of the 94 top genes, and the regulatory relationship between the 21 TFs and the 94 top genes was visualized using Cytoscape 3.8.2 software (Cytoscape Consortium, San Diego, CA, USA) in Fig. 6a. Nevertheless, during the alignment process, genes in *K. marxianus* annotated with the same symbol as in *S. cerevisiae* were found to differ in the patterns of TFs binding in the promoter region, which indicates that these two yeast species may have different regulation networks.

Gcr1 (Hossain et al. 2016), a critical TFs regulating glycolysis, was upregulated by SA treatment (Fig. 6a), which is consistent with our previous analysis that energy metabolism is an important SA response. Nrg1 (a negative regulator of glucose-repressed genes) was downregulated, which could explain the enhancement of glycolysis under SA stress (Zhou and Winston 2001). TF Upc2 which promotes lipid and sterol biosynthesis was upregulated (Jorda and Puig 2020), which may explain the significant enrichment of the sterol synthesis up-regulation in the GO analysis mentioned above. The TF Ndt80 induces the expression of middle meiosis genes required for the meiotic divisions (Tsuchiya et al. 2014).

To further explore which TFs could enhance the SA tolerance, we overexpressed these candidate genes with the episomal plasmids pUTCP2 (Supplementary Fig. S2) derived from plasmid pUKDN132 marked with the 5-phosphate orotate gene (Zhou et al. 2018). A series of plasmids and strains were obtained (Supplementary Table S1). The candidate TFs and genes were overexpressed under the control of the *IMTCP2* promoter. All the recombinant strains (from line 43 to line 47 of Supplementary Table S1) were cultured in 20 g/L (growth of strains totally inhibited under 30) SA to test the tolerance. From Fig. 6b and c, we found that overexpression of TFs Gcr1, Upc2, and Ndt80 on an episomal plasmid could significantly increase the biomass 88.7%, 70.3% and 41.7% in strain ZW01 (NBRC1777, *ura3∆*) compared with the strain harboring empty plasmid as the control, respectively.

## Discussion

*K. marxianus* has become an attractive non-conventional yeast chassis cell factory due to its excellent industrial properties (Qiu et al. 2023). Among various products that can be produced by *K. marxianus*, organic acids have received increasing interest, especially as monomers of bioplastics (Zhang et al. 2023). However, response to different SA concentrations and SA toxicity in *K. marxianus* has not been reported. One of the key challenges for acid production is the toxicity of the products. On the other hand, global response to different concentrations of SA is of importance for further engineering of strains with dynamic control of gene expression. In this work, global gene transcription changes of *K. marxianus* exposed to various concentrations of SA were analyzed. Enrichment and analysis of interested gene clusters revealed repression of the TCA cycle and glyoxylate cycle, as well as activation of the glycolysis pathway and genes related to ergosterol synthesis. Meanwhile, promoters responding to SA were investigated and TFs increasing SA tolerance were identified.

Compared to the single concentration of SA to perturbed cell global gene transcription, the analysis of four different SA concentrations (Fig. 1) could increase the probability of screening for the desired target genes. Previous studies on responses of *K. marxianus* to various conditions (e.g. high temperature, ethanol) used only drastic levels of treatments (Diniz et al. 2017; Kosaka et al. 2022) or different growth stages (Yu et al. 2021). Our studies are different in that gradually increased concentrations were employed. Therefore, differently from the previous studies, we classified genes into different response patterns under SA stress in *K. marxianus* by integrating the data from all the genes at the four concentrations (Fig. 2), no matter whether these genes showed significant changes or not. By this method, a more global landscape of gene expression can be revealed and provide a strategy for studying metabolite response mechanisms across various organisms.

In a previous study, both genes related to the TCA cycle and involved in the glycolysis pathway were downregulated when the *K. marxianus* strain was subjected to the 6% ethanol stress (Diniz et al. 2017). In another study, under the multiple inhibitors’ stresses (including acetic acid, phenols, furfural, and 5-hydroxymethylfurfural), the TCA cycle related genes were upregulated and the glycolysis pathway related genes downregulated (Wang et al. 2018). However, in this case, the TCA cycle related genes were downregulated and the glycolysis related genes were upregulated when the strains were exposed to the SA stress (Fig. 4), which implied the different response patterns when the strains faced different stresses. Meanwhile, many genes of the glycolysis pathway were upregulated, which implied that the enhanced glycolytic pathway provides a faster rate of ATP supply when the TCA cycle is inhibited by SA, an intermediate product of the TCA cycle. These speculations are also consistent with the observed increased capacity of per OD_600_ cells to produce ethanol (Supplementary Fig. S3a).

The typical promoter consists of the core promoter and upstream activation sequences (UASs), where binding TFs can promote the target genes to respond to inducers, such as acetic acid (Erden-Karaoglan and Karaoglan 2022; Yan et al. 2022; Kim et al. 2019). Accordingly, screening the involved promoters can be used as a potential biosensor that specifically responds to SA or applied as a dynamic regulation tool in metabolic engineering under different SA concentrations, its transcription strength alteration including enhanced or weakened. In this case, the promoters of genes *IMTCP2* and *KLMA_50123* exhibited significant characteristics of SA response (Fig. 5c, 5d). Then, the candidate SA-responsive TFs could be narrowed down by truncating the promoter length until the SA-responsive phenomenon disappeared (Cazier and Blazeck 2021; Lang et al. 2020; Zhang et al. 2018). SA is not only a metabolic intermediate but also a signal molecule that could regulate muscle remodeling in response to exercise in mammals, stabilize hypoxia inducible factor-1α (HIF-1α) by inhibiting prolyl hydroxylase and lead to cancer cell migration (Reddy et al. 2020). SA increases the expression of succinate receptor-1 (SUCNR-1) in cancer cells, which is considered a target for the development of new anti-metastasis drugs. In addition, serum succinate which is elevated in cancer patients may become a theranostic biomarker for medical diagnosis (Kuo et al. 2022). Our study showed that yeast cells respond to various concentrations of SA, and it is of interest to further study whether SA is a signal molecule for microbial metabolism.

Notably, the alteration of transcription was regulated by the TFs binding with the cis-acting elements or interaction between TFs. So, the top 100 genes regulation network was listed including their prediction factors investigated in transcription regulation (Fig. 6a). Among the regulators, Gcr1, Upc1, and Hap1 activate glycolysis pathway, ergosterol biosynthesis and respond to heme and oxygen related genes, respectively (Hickman and Winston 2007; Jorda and Puig 2020). We revealed that overexpression of *GCR1* and *UPC2* improved the resistance to SA in chassis host *K. marxianus*, which is helpful for the development of SA producers. A recent report shows that high malic acid resistance is beneficial for the production of high concentration of malic acid (Sun et al. 2023). Therefore, the SA tolerant strain we developed in this study may serve as an optimal parent strain for SA production.

In summary, the transcriptome analysis in this study revealed the global response of *K. marxianus* to SA. We found that SA treatment enhanced the glycolytic pathway that inhibited the TCA cycle, and revealed new candidate target genes including coding TFs, and SA respond promoter biological elements used in metabolic engineering. This work not only benefits understanding the mechanism of SA response but also provides a reference for developing organic acid tolerant yeast.

## Supplementary Information

Below is the link to the electronic supplementary material.

Supplementary file1 (PDF 472 KB)

Supplementary file2 (XLSX 1198 KB)

## Data Availability

The combined fast files containing the sequenced reads have been uploaded in Sequence Read Archive (SRA) of NCBI and associated link at the below https://dataview.ncbi.nlm.nih.gov/object/PRJNA1017639?reviewer=4n0lqca1kms0f94le9pi9k4rin.
