# Supplementary material for: Transcriptome analysis of Kluyveromyces marxianus under succinic acid stress and development of robust strains
Source: Appl Microbiol Biotechnol. 2024 Apr 9;108:293. doi: 10.1007/s00253-024-13097-3 (PMC11003901; doi:10.1007/s00253-024-13097-3)
Supplement: Supplementary file 1 [file 253_2024_13097_MOESM1_ESM.pdf]

Supplementary materials

Applied Microbiology and Biotechnology

Transcriptome analysis of *Kluyveromyces marxianus* under succinic acid stress and development of a robust strain

Du-Wen Zeng[a], Yong-Qiang Yang[c], Qi Wang[a], Feng-Li Zhang[a], Mao-Dong Zhang[a], Sha Liao[b], Zhi-Qiang Liu[c], Ya-Chao Fan[b], Chen-Guang Liu[a], Lin Zhang[b*], Xin-Qing Zhao[a*]

[a]Key Laboratory of Microbial Metabolism, Joint International Research Laboratory of Metabolic & Developmental Sciences, School of Life Sciences and Biotechnology, Shanghai Jiao Tong University, Shanghai 200240, China

[b]SINOPEC Dalian Research Institute of Petroleum and Petrochemicals Co., Ltd, Dalian 116045, China

[c]School of Life Sciences, Hainan University, Haikou 570228, China

[*]Corresponding authors: Lin Zhang, e-mail address, zhl.fshy@sinopec.com, phone number, +86-0411-396969806; Xin-Qing Zhao, e-mail address, xqzhao@sjtu.edu.cn, phone number, +86-021-34206673.

Construction of plasmids and strains

For the construction of plasmids, pUCC001 (Rajkumar and Morrissey 2022) was utilized as the initial plasmid (Table S1). The backbone of the plasmid, which included the hygromycin resistance cassette, was obtained by removing the Cas9 expression cassette via *Bst*EII and *Afl*II digestion. The expression cassettes of the fluorescent protein reporter genes (*yeGFP* and *mcherry*) were ligated to the plasmid backbone in the following steps. Firstly, the fragments *IMTCP2*p, *yeGFP*, and *IMTT1*t were amplified from the *K. marxianus* NBRC1777 genome and plasmid pKT0209 (Sheff and Thorn 2004) using the primer pairs from Table S2. Then, the expression cassette for green fluorescent protein (*IMTCP2*p-*yeGFP*-*IMTT1*t) was created using the overlap extension polymerase chain reaction (OE-PCR) technique. This cassette was then ligated to the plasmid skeleton between the *Bst*EII and *Afl*II sites by Gibson assembly to obtain plasmid pUCZ01 (see Table S1, line 2). Similarly, the red fluorescent protein mcherry (donated from Prof. Xie at Shanghai jiaotong University) expression cassette comprises promoter sc*TDH3* (glyceraldehyde 3-phosphate dehydrogenase), *mcherry*, and sc*CYC1* terminator to obtain plasmid pUCZ02 (see Table S1, line 3). This cassette was ligated to the skeleton of plasmid pUCZ01 after digested with *Bsm*BI for removing the originally existed guide RNA expression cassette and the plasmid cyclized by Gibson assembly to obtained plasmid pUCZ03 (Table S1, line 4). Subsequently, the hygromycin marker of pUCZ03 was removed after digestion by *Psp*XI and *Bam*HI, then replaced by gene *URA3* coding orotidine-5'-phosphate decarboxylase amplified from NBRC1777 genome between the *Psp*XI and *Bam*HI sites to obtain the plasmid

pUCZ04 (Table S1, line 5). Finally, a series of plasmids (from Table S1 line 5 to line 17) derived from pUCZ04 by replacing the original promoter *IMTCP2*p of *yeGFP*. The plasmid pUKDN132 (Zhou et al. 2018) removed the original promoter and signal peptide sequences and replaced by fragment of the *IMTCP2* promoter through digestion of *Sac*II and *Not*I to obtain the episomal plasmid pUTCP2, which was used as the vector for the overexpression of transcription factors (Fig. S2).

The *URA3* gene encoding orotidine-5'-phosphate decarboxylase was disrupted in *K. marxianus* NBRC1777 to obtain strain ZW01 by the CRISPR/Cas9-mediated genome editing method (Rajkumar et al. 2019). Yeast transformation was performed using the lithium acetate transformation method (Lyu et al. 2021).

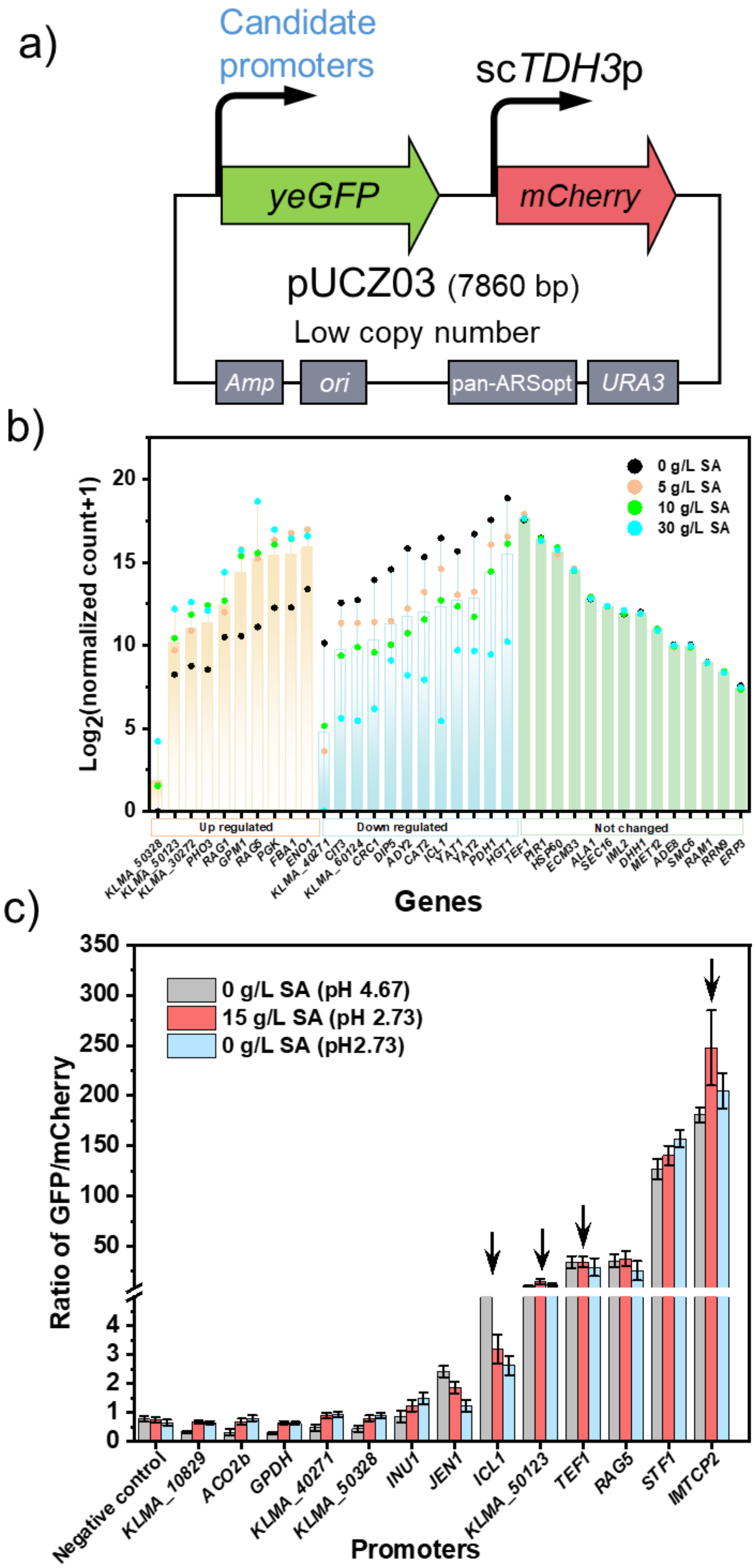
a)
Candidate promoters
scTDH3p
yeGFP
mCherry
pUCZ03 (7860 bp)
Low copy number
Amp
ori
pan-ARSopt
URA3
b)
Log2(normalized count+1)
0 g/L SA
5 g/L SA
10 g/L SA
30 g/L SA
Up regulated
Down regulated
Not changed
KLMA_50328
KLMA_50123
KLMA_30272
PHO3
RAG1
GPM1
RAG5
PGK
FBA1
ENO1
KLMA_40271
CIT3
KLMA_60124
CRC1
DIP5
ADY2
CAT2
ICL1
YAT1
YAT2
PDH1
HGT1
TEF1
PIR1
HSP60
ECM33
ALA1
SEC16
IML2
DHH1
MET12
ADE8
SMC6
RAM1
RRN9
ERP3
Genes
c)
Ratio of GFP/mCherry
0 g/L SA (pH 4.67)
15 g/L SA (pH 2.73)
0 g/L SA (pH2.73)
Negative control
KLMA_10829
ACO2b
GPDH
KLMA_40271
KLMA_50328
INU1
JEN1
ICL1
KLMA_50123
TEF1
RAG5
STF1
IMTCP2
Promoters

Figure S1 The selection and verification of promoters responding to SA. (a) The candidate SA-responsive promoter verification by the double fluorescence expression system on low copy plasmid pUCZ03, in which the candidate promoters and sc*TDH3* control the green and red fluorescent protein expression respectively. (b) The relative transcription level of a gene under different concentrations of SA selected from transcriptome data. The x-axis divided into three types including upregulated (orange bars), downregulated (blue bars) and not changed (green bars), in which the ordinate values of the small balls (black, yellow, green and blue) represent the transcription levels of a candidate gene under the 0, 5, 10, and 30 g/L SA, respectively. (c) Ratio of green and red fluorescent intensity at 15 g/L SA (pH 2.73) or without SA but with pH adjustment to 2.73 by hydrochloric acid.

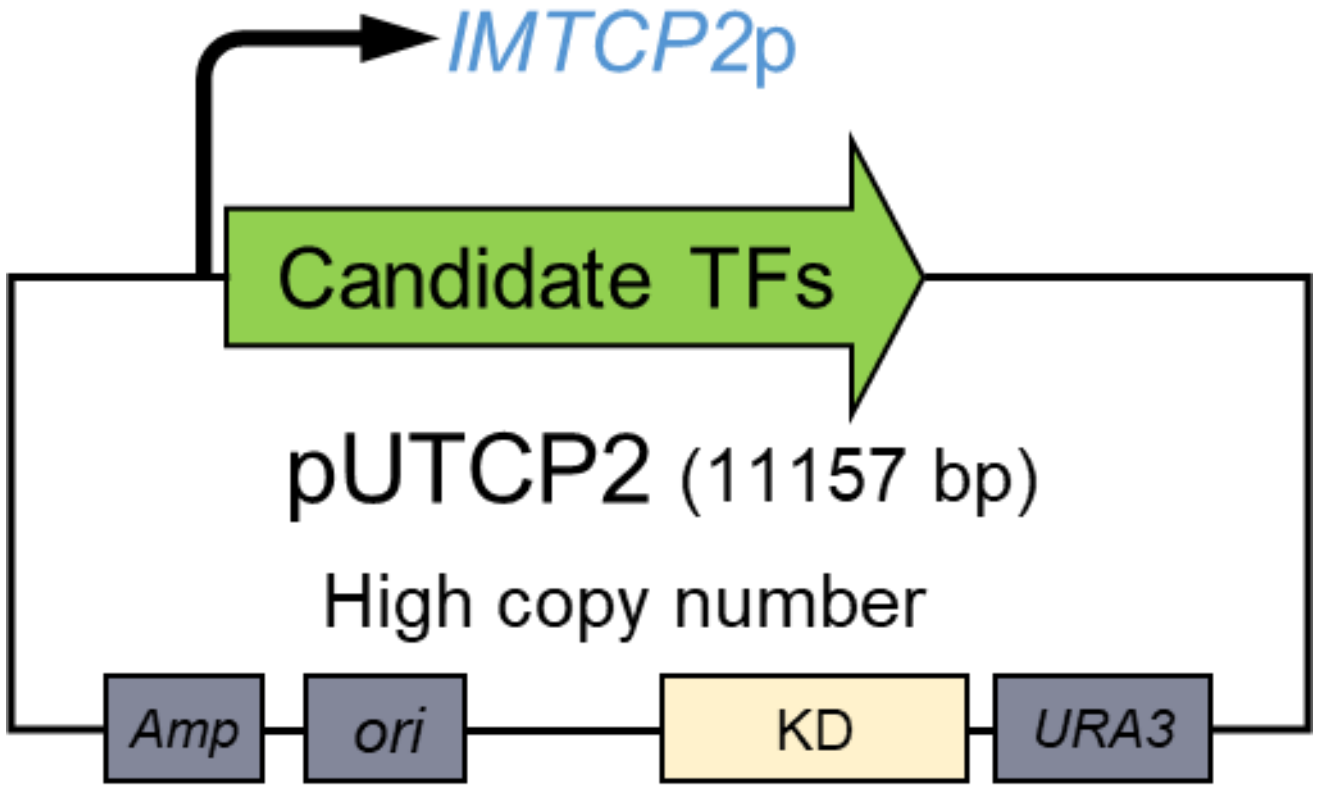

Figure S2 Transcription factors overexpression system, which is under the control of promoter *IMTCP2* on the high copy number plasmid pUTCP2 with *URA3* as selected marker.

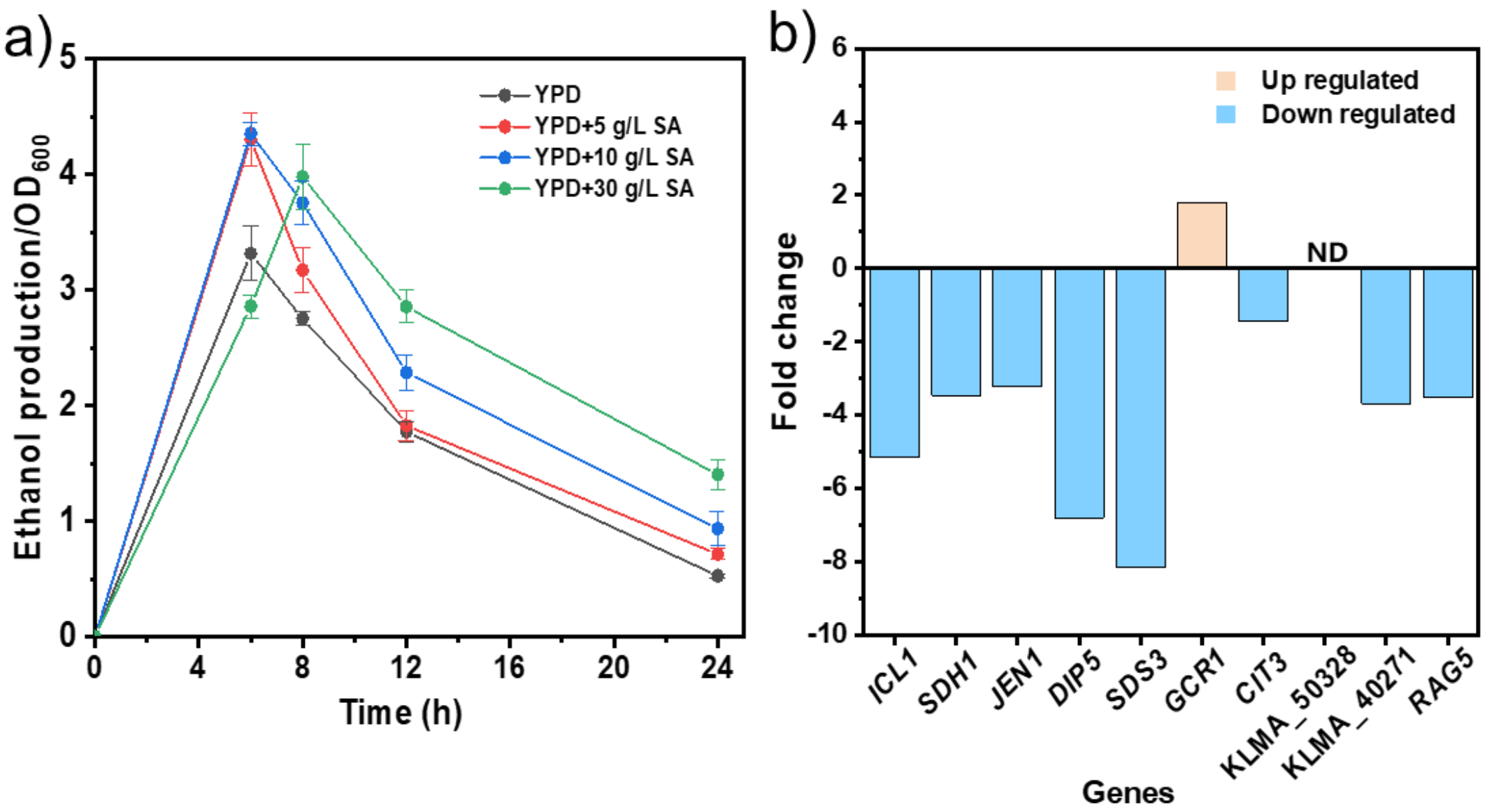

Figure S3 The glycolytic pathway was enhanced and the tricarboxylic acid cycle was repressed under SA stress. (a) The ethanol production capacity of per OD600 cells of NBRC1777 was improved under the different concentrations of SA including 0, 5, 10, and 30 g/L in YPD medium cultured in 37 ºC and 200 rpm for 24 h. (b) Verification of transcription level change of key genes selected from transcriptome data by qPCR under 30 g/L SA in YPD medium cultured in 37 ºC and 200 rpm for 6 h.

Table S1 Plasmids and strains

Plasmids	Genotype	Resource
(1) pUCC001	Centromeric plasmid; *HygR*, pan-ARS, (Beta-galactosidase)-gRNA scaffold, *CAS9*	(Rajkumar and Morrissey 2022)
(2) pUCZ01	pUCC001 derivative; *KmURA3*, pan-ARSopt, *IMTCP2*p-*IMTT1*t	This study
(3) PUCZ02	pUCZ01 derivative; *KmURA3*, pan-ARSopt, *IMTCP2*p-*yeGFP-IMTT1*t	This study
(4) pUCZ03	pUCZ02 derivative; *KmURA3*, pan-ARSopt, *scTDH3*p- *mCherry* -*CYC1*t	This study
(5) pUCZ04	pUCZ03 derivative; *KmURA3*, pan-ARSopt, *IMTCP2*p-*yeGFP-IMTT1*t, *scTDH3*p-*mCherry*-*CYC1*t	This study
(6) pUCZ04-INU1	pUCZ04 derivative; *KmURA3*, pan-ARSopt, *INU1*p-*yeGFP-IMTT1*t, *scTDH3*p-*mCherry*-*CYC1*t	This study
(7) pUCZ04-TEF1	pUCZ04 derivative; *KmURA3*, pan-ARSopt, *TEF1*p-*yeGFP-IMTT1*t, *scTDH3*p-*mCherry*-*CYC1*t	This study
(8) pUCZ04-JEN1	pUCZ04 derivative; *KmURA3*, pan-ARSopt, *JEN1*p-*yeGFP-IMTT1*t, *scTDH3*p-*mCherry*-*CYC1*t	This study
(9) pUCZ04-K50328	pUCZ04 derivative; *KmURA3*, pan-ARSopt, *KLMA_50328*p-*yeGFP-IMTT1*t, *scTDH3*p-*mCherry*-*CYC*t	This study
(10) pUCZ04-ICL1	pUCZ04 derivative; *KmURA3*, pan-ARSopt, *ICL1*p-*yeGFP-IMTT1*t, *scTDH3*p-*mCherry*-*CYC1*t	This study
(11) pUCZ04-K40271	pUCZ04 derivative; *KmURA3*, pan-ARSopt, *KLMA_40271*p-*yeGFP-IMTT1*t, *scTDH3*p-*mCherry*-*CYC1*t	This study
(12) pUCZ04-RAG5	pUCZ04 derivative; *KmURA3*, pan-ARSopt, *RAG5*p-*yeGFP-IMTT1*t, *scTDH3*p-*mCherry*-*CYC1*t	This study
(13) pUCZ04-STF2	pUCZ04 derivative; *KmURA3*, pan-ARSopt, *STF2*p-*yeGFP-IMTT1*t, *scTDH3*p-*mCherry*-*CYC1*t	This study
(14) pUCZ04-Aco2b	pUCZ04 derivative; *KmURA3*, pan-ARSopt, *Acocb*p-*yeGFP-IMTT1*t, *scTDH3*p-*mCherry*-*CYC1*t	This study
(15) pUCZ04-K10829	pUCZ04 derivative; *KmURA3*, pan-ARSopt, *KLMA_10829*p-*yeGFP-IMTT1*t, *scTDH3*p-*mCherry*-*CYC1*t	This study
(16) pUCZ04-GPDH	pUCZ04 derivative; *KmURA3*, pan-ARSopt, *GPDH*p-*yeGFP-IMTT1*t, *scTDH3*p-*mCherry*-*CYC1*t	This study
(17) pUCZ04-K50231	pUCZ04 derivative; *KmURA3*, pan-ARSopt, *KLMA_50123*p-*yeGFP-IMTT1*t, *scTDH3*p-*mCherry*-*CYC1*t	This study
(18) pUKDN132	Episomal plasmid; *KmURA3*, KD, *INU1*p-(INU1 signal peptide)-*INU1*t	(Zhou et al. 2018)
(19) pUTCP2	pUTCP2 derivative; *KmURA3*, KD, *IMTCP2*p-*INU1*t	This study
(20) pUTCP2-GCR1	pUTCP2 derivative; *KmURA3*, KD, *IMTCP2*p-*GCR1-INU1*t	This study
(21) pUTCP2-UPC2	pUTCP2 derivative; *KmURA3*, KD, *IMTCP2*p-*UPC2-INU1*t	This study
(22) pUTCP2-NRG1	pUTCP2 derivative; *KmURA3*, KD, *IMTCP2*p-*NRG1-INU1*t	This study
(23) pUTCP2-NDT1	pUTCP2 derivative; *KmURA3*, KD, *IMTCP2*p-*NDT1-INU1*t	This study
Strains		

K. marxianus		
(24) NBRC1777	Wild type, haploid	NBRC
(25) ZW01	Derived from NBRC1777, *ura3*Δ	This study
(26) NBKD	Derived from NBRC1777, *ku70*Δ :: *KmIMTCP1*p-*scRAD52-TIMTT2*t	This study
(27) ZW01-none	ZW01 derivative; empty plasmid {pUCZ01}/(*KmURA3*)	This study
(28) ZW01-gfp	ZW01 derivative; plasmid {pUCZ02}/(*KmURA3*, *IMTCP2*p-*yeGFP*)	This study
(29) ZW01-mch	ZW01 derivative; plasmid {pUCZ03}/(*KmURA3*, *mCherry*)	This study
(30) ZW01-TCP2p	ZW01 derivative; plasmid {pUCZ04}/(*KmURA3*, *mCherry*, *IMTCP2*p-*yeGFP*)	This study
(31) ZW01-INU1p	ZW01 derivative; plasmid {pUCZ04-INU1}/(*KmURA3*, *mCherry*, *INU1*p-*yeGFP*)	This study
(32) ZW01-TEF1p	ZW01 derivative; plasmid {pUCZ04-TEF1}/(*KmURA3*, *mCherry*, *TEF1*p-*yeGFP*)	This study
(33) ZW01-JENp	ZW01 derivative; plasmid {pUCZ04-JEN1}/(*KmURA3*, *mCherry*, *JEN1*p-*yeGFP*)	This study
(34) ZW01-K50328p	ZW01 derivative; plasmid {pUCZ04-K50328}/(*KmURA3*, *mCherry*, *KLMA_50328*p-*yeGFP*)	This study
(35) ZW01-ICL1p	ZW01 derivative; plasmid {pUCZ04-ICL1}/(*KmURA3*, *mCherry*, *ICL1*p-*yeGFP*)	This study
(36) ZW01-K40271p	ZW01 derivative; plasmid {pUCZ04-K40271}/(*KmURA3*, *mCherry*, *KLMA_40271*p-*yeGFP*)	This study
(37) ZW01-RAG5p	ZW01 derivative; plasmid {pUCZ04-RAG5}/(*KmURA3*, *mCherry*, *RAG5*p-*yeGFP*)	This study
(38) ZW01-STF2p	ZW01 derivative; plasmid {pUCZ04-STF2}/(*KmURA3*, *mCherry*, *STF2*p-*yeGFP*)	This study
(39) ZW01-Aco2bp	ZW01 derivative; plasmid {pUCZ04-Aco2b}/(*KmURA3*, *mCherry*, *Aco2b*p-*yeGFP*)	This study
(40) ZW01-K10829p	ZW01 derivative; plasmid {pUCZ04-K10829}/(*KmURA3*, *mCherry*, *KLMA_10829*p-*yeGFP*)	This study
(41) ZW01-GPDHp	ZW01 derivative; plasmid {pUCZ04-GPDH}/(*KmURA3*, *mCherry*, *GPDH*p-*yeGFP*)	This study
(42) ZW01-K50231p	ZW01 derivative; plasmid {pUCZ04-K10829}/(*KmURA3*, *mCherry*, *KLMA_50231*p-*yeGFP*)	This study
(43) ZW01-TCP2	ZW01 derivative; plasmid {pUTCP2}/(*KmURA3*, *KD*)	This study
(44) ZW01-GCR1	ZW01 derivative; plasmid {pUTCP2-GCR1}/(*KmURA3*, *KD*, *GCR1*)	This study
(45) ZW01-UPC2	ZW01 derivative; plasmid {pUTCP2-UPC2}/(*KmURA3*, *KD*, *UPC2*)	This study
(46) ZW01-NRG1	ZW01 derivative; plasmid {pUTCP2-NRG1}/(*KmURA3*, *KD*, *NRG1*)	This study
(47) ZW01-NDT1	ZW01 derivative; plasmid {pUTCP2-NDT1}/(*KmURA3*, *KD*, *NDT1*)	This study

Table S2 Primers used in this study

Primer name	Sequence (5'→3')	Purpose
BstE2-sbf1-TCP2-F	CCGGGTAACCCCTGCAGGCAAAACAGGACAAAACAAAACAATACAGTAC	Amplification of *IMTCP2* promoter
TCP2-eGFP-R	AATTCTTCACCTTTAGACATGTCGACTTTTGATTTGTGTTTAAGCGAGTGACTG	
eGFP-TCP2-F	CTTAAACACAAATCAAAAGTCGACATGTCTAAAGGTGAAGAATTATTCACTGG	Amplification of *yeGFP*
eGFP-MTT1-R	GATTTTATCAACAAAGCTTCTCTAGATTATTTGTACAATTCATCCATACCATGGG	
MTT1-F	TCTAGAGAAGCTTTGTTGATAAAATCTAACTACTG	Amplification of *IMTT1* terminator
Alf2-Asc1-MTT1-R	GGGCTTAAGGGCGCGCCGTTCAAATACGGAAGCAAAGGAAC	
scTDH3p-F	CAAATCATAATCAGCACTAACGTCTCATCATTATCAATACTGCCATTTCAAAGAATACG	Amplification of sc*TDH3* promoter
scTDH3-R	TTTGTTTGTTTATGTGTGTTTATTCGAAACTAAG	
mCherry-F	CGAATAAACACACATAAACAAACAAAATGGTTTCTAAGGGTGAAGAAGAC	Amplification of mCherry
mCherry-R	ACAAAGGAAAAGGGGCCTGTTTACTTATACAATTCATCCATACCACCAG	
tCYC1-F	ACAGGCCCCTTTTCCTTTGTC	Amplification of *CYC1* terminator
tCYC1-R	CTTAGATTGTCGCTACGGCATATACGAGAGACGGTCCCAAAACCTTCTCAAGCAAGG	
km50123-F	AGGACGACCGGGTAACCCCTGCAGGTATTCTTCCTCCCTTGCTATTCTTTC	Amplification of KLMA_50123 promoter
km50123-R	GAATAATTCTTCACCTTTAGACATGTCGACGATTACTGTGTTATTTTTCGATTCG	
Sbf1-50328p-F	AGGACGACCGGGTAACCCCTGCAGGCTTACTCTGCCTGCTGAGCTTG	Amplification of KLMA_50328 promoter
SalI-50328p-R	GAATAATTCTTCACCTTTAGACATGTCGACGATTACTGTGTTATTTTTCGATTCG	
Sbf1-ICL1p-F	AGGACGACCGGGTAACCCCTGCAGGCTACGTCATGAGTCTGCCAT	Amplification of *ICL1* promoter
SalI-ICL1p-R	AATAATTCTTCACCTTTAGACATGTCGACTCTTCTTCTTGAATATTGTTCGTTTGTATG	
Sbf1-INUIp-F	AGGACGACCGGGTAACCCCTGCAGGGTTGCAAGTTGCACGCTGGA	Amplification of *INU1* promoter
salI-INU1p-R	AATAATTCTTCACCTTTAGACATGTCGACATCTAACAAAAAAAAAATTAAATGTGTCAC	
SbfI-JEN1p-F	GGACGACCGGGTAACCCCTGCAGGGGCTTGGATGGAAACTCGCC	Amplification of *JEN1* promoter

SalI-JEN1p-R	GAATAATTCTTCACCTTTAGACATGTCGACTATGCCTATAGTAGCTTGTGTATTAGCTG	
Sbf1-TEFp-F	AGGACGACCGGGTAACCCCTGCAGGAGATTAAAAAAAAAGTACAGTTAGTTAGAGCAGG	Amplification of *TEF1* promoter
SalI-TEFp-R	GTGAATAATTCTTCACCTTTAGACATGTCGACCTTTAATGTTACTTCTCTTGGAGTTAG	
Sbf1-40271p-F	AGGACGACCGGGTAACCCCTGCAGGGGTAACACAGAACTCTGACTCTCC	Amplification of KLMA_40271 promoter
Sal1-40271p-R	GAATAATTCTTCACCTTTAGACATGTCGACGGCAATCCTGTTCTCATACCTGATG	
STF2p-F	AGGACGACCGGGTAACCCCTGCAGGGACAAAGTCCAATGCCTCTC	Amplification of *STF2* promoter
STF2p-R	AATTCTTCACCTTTAGACATGTCGACGATGTAATGTAGTATTTGTTGTGTAAATG	
Sbf1-ACO2bp-F	CAAAGGACGACCGGGTAACCCCTGCAGGAACTTGCTCCGATCCTCGGA	Amplification of *ACO2b* promoter
Sbf1-ACO2bp-R	GTGAATAATTCTTCACCTTTAGACATGTCGACTATCAACAGATGATATGGTTTG	
Sbf1-GPDHp-F	AGGACGACCGGGTAACCCCTGCAGGAAGCGTCTCGTCAAGGACCG	Amplification of *GPDH* promoter
Sbf1-GPDHp-R	ATAATTCTTCACCTTTAGACATGTCGACCTTTTCTAACTACTACGCAATTGCTGCTG	
Sbf1-RAG5p-F	AGGACGACCGGGTAACCCCTGCAGGCCGAGTCCTACGCCAGCTAC	Amplification of *RAG5* promoter
Sbf1-RAG5p-R	AATAATTCTTCACCTTTAGACATGTCGACTTTTGTAAGTGTGTGTGTTTGTAATAATTG	
Sbf1-km10829-F	AGGACGACCGGGTAACCCCTGCAGGAATTGAAAACACTATTAAAATTTGTTGTAAGACC	Amplification of KLMA_10829 promoter
Sbf1-km10829-R	GTGAATAATTCTTCACCTTTAGACATGTCGACGTCTGCTAAAAGTTCAAAATTAATTCG	
KD-pTCP2-F	ATTACCGTGCCGATTCGCACGCTGCAACCGCGGCAAAACAGGACAAAACAAAACAATACAG	Amplification of *IMTCP2* promoter
INUt-IMTCP2-R	GATCAGATCAAAGCTTGCGGCCTTAAGCGGCCGCACTAGTCCTGCAGGTTTTGATTTGTGTTTAAGCGAGTGACTG	
TCP2-UPC2-F	CTTCAGTCACTCGCTTAAACACAAATCAAAACCTGCAATGAGTACAGAAAGAATGCAATCTTC	Amplification of *UPC2* promoter
UPC2-R	GCTTGCGGCCTTAAGCGGCCGCACTAGTCCTGCACTAGAAAATTCCAAAATCCGACAAGTTTG	
TCP2-NRG1-F	TCAGTCACTCGCTTAAACACAAATCAAAACCTGCAATGAGTATTGTTCGCCCAAATATG	Amplification of *NRG1* promoter
NRG1-R	GCTTGCGGCCTTAAGCGGCCGCACTAGTCCTGCATCATGTGTCGATTGGAGGTG	

TCP2-NDT80-F	CTTCAGTCACTCGCTTAAACACAAATCAAAACCTGCAATGATATCTACTCTGCAGCAGTTTATTG	Amplification of *NDT80* promoter
TCP2-NDT80-R	GCGGCCTTAAGCGGCCGCACTAGTCCTGCATTAGTGCCGGAATATGCTCG	
CP2-GCR1-F	ACTCGCTTAAACACAAATCAAAACCTGCAATGGACAAAGACAATTTGAATATCAATTTG	Amplification of *GCR1* promoter
CP2-GCR1-R	CGGCCTTAAGCGGCCGCACTAGTCCTGCATTATTCATTATCATCTTGCGAGTAATTATC	
pIMTCP1-F	TGCGGAGGTCTCCTACGCTTGTTTCTCCATTAGGATC	*IMTCP1* promoter
pIMTCP1-R	TCCGCAGGTCTCGTCATTTTTAACTTCTTTGTGTTGGTTTTTTAGGC	
G-URA3△-F	GAGGACGAAACGAGTAAGCTCGTCTGGTTTGAAGCAAGGTGCCGGTTTTAGAGCTAGAAATAGCAAG	gRNA of *URA3* deletion
G-URA3△-R	CTTGCTATTTCTAGCTCTAAAACCGGCACCTTGCTTCAAACCAGACGAGCTTACTCGTTTCGTCCTC	
Dor_ura3_up1000-F	TTAAGTAGTCAAACAAATTGTGTTGAAAAG	Donor DNA of *URA3*
Dor_ura3_dn1000-R	GTCTGCAACACCGATTTATCGG	
ACT-F	ACGGTATCGTTACCAACTGGG	For qPCR
ACT-R	GGGGCTTCGGTCAACAAAAC	
ICL1-F	TTCTTCGACTGGGACTTGCC	For qPCR
ICL1-R	TCGACTCCATCCAGCAGAGA	
SDH1-F	CCCGTGAAGCTCCAAAGTCT	For qPCR
SDH1-R	ATGCTTGACCACCCTTACCG	
JEN1-F	GCCATGGGTGGTATCTACGG	For qPCR
JEN1-R	TTGACGTGCAAGAAGGCTCT	
DIP5-F	GTATCCCAGTGCTCCCATCG	For qPCR
DIP5-R	CGATAGCCAAGCCGTAGAGG	
SDS3-F	CAAAGCTGAACACGAACGCA	For qPCR
SDS3-R	AAGATTGAGCGTTTGCGACG	
GCR1-F	GCCGTAACTGCAGAAGGTCT	For qPCR
GCR1-R	AGTTAATGGCTGCTTGGGGG	
CIT3-F	ACGTTTCTGCCCACACTACC	For qPCR
CIT3-R	CTTCTTGTGCCGCTAAACCG	
KLMA_50328-F	GAAACCGATCCTACCTGCGT	For qPCR
KLMA_50328-R	TGCAGGCCTCTTAGAATCGC	
KLMA_40271-F	GTACGGTGTCTCCGGAATG	For qPCR
KLMA_40271-R	GAACCCTCGGGGTATTGCTC	
RAG5-F	CACCTACCCAGCCAAGATCG	For qPCR
RAG5-R	TGTTCCGACCAATTCAGCCA	

Reference for the supplementary materials

Lyu Y, Wu P, Zhou J, Yu Y, Lu H (2021) Protoplast transformation of *Kluyveromyces marxianus*. Biotechnol J 16(12):e2100122. doi:10.1002/biot.202100122

Rajkumar AS, Morrissey JP (2022) Protocols for marker-free gene knock-out and knock-down in *Kluyveromyces marxianus* using CRISPR/Cas9. FEMS Yeast Res 22(1):foab067. doi:10.1093/femsyr/foab067

Rajkumar AS, Varela JA, Juergens H, Daran JG, Morrissey JP (2019) Biological parts for *Kluyveromyces marxianus* synthetic biology. Front Bioeng Biotechnol 7(97):97. doi:10.3389/fbioe.2019.00097

Sheff MA, Thorn KS (2004) Optimized cassettes for fluorescent protein tagging in *Saccharomyces cerevisiae*. Yeast 21(8):661-670. doi:10.1002/yea.1130

Zhou J, Zhu P, Hu X, Lu H, Yu Y (2018) Improved secretory expression of lignocellulolytic enzymes in *Kluyveromyces marxianus* by promoter and signal sequence engineering. Biotechnol Biofuels 11:235. doi:10.1186/s13068-018-1232-7
